# Introducing voluntary private health insurance in a mixed medical economy: are Hong Kong citizens willing to subscribe?

**DOI:** 10.1186/s12913-017-2559-7

**Published:** 2017-08-25

**Authors:** Alex Jingwei He

**Affiliations:** Department of Asian and Policy Studies, The Education University of Hong Kong, 10 Lo Ping Road, Tai Po, New Territories Hong Kong

**Keywords:** Voluntary health insurance, Health financing, Health policy, Hong Kong

## Abstract

**Background:**

Struggling to correct the public-private imbalance in its health care system, the Hong Kong SAR Government seeks to introduce a government-regulated voluntary health insurance scheme, or VHIS, a distinctive financing instrument that combines the characteristics of private insurance with strong government regulation. This study examines citizens’ responses to the new scheme and their willingness to subscribe.

**Methods:**

First-hand data were collected from a telephone survey that randomly sampled 1793 Hong Kong adults from September 2014 to February 2015. Univariate and multivariate methods were employed in data analysis.

**Results:**

More than one third of the respondents explicitly stated intention of subscribing to the VHIS, a fairly high figure considering the scheme’s voluntary nature. Multivariate analysis revealed moderate evidence of adverse selection, defined as individuals’ opportunistic behaviors when making insurance purchasing decision based on their own assessment of risks or likelihood of making a claim.

**Conclusion:**

The excellent performance of Hong Kong’s public medical system has had two parallel impacts. On the one hand, high-risk residents, particularly the uninsured, do not face a pressing need to switch out of the overloaded public system despite its inadequacies; this, in turn, may reduce the impact of adverse selection that may lead to detrimental effects to the insurance market. On the other hand, high satisfaction reinforces the interests of those who have both the need for better services and the ability to pay for supplementary insurance. Furthermore, the high-risk population demonstrates a moderate interest in the insurance despite the availability of government subsidies. This may offset the intended effect of the reform to some extent.

## Background

The role of private health insurance has been gradually recognized in the global search for viable sources of health care financing. Despite being traditionally associated with notions of unequal access, unaffordability, adverse selection, cream-skimming, and elite care for the rich, it is increasingly seen in many health systems as a useful tool for achieving a wide range of policy goals [1, 2]. The advantages of private health insurance for high-income countries lie predominantly on greater consumer choice, mobilizing additional resources, enhanced individual responsibility, meeting the needs of the upper and middle classes, market competition, and better cost control [3]. While private health insurance represents a much smaller share of the total health funding in high-income countries, compared to social insurance and taxation, it plays a variety of roles ranging from primary coverage for particular subgroups to a supporting role for public systems [4]. In one third of the Organization for Economic Co-operation and Development (OECD) countries, private insurance covers approximately 30% of the population, making it an important source of health financing [5].

Private health insurance plays a relatively minor role in Asia [6]. While most industrialized Asian economies, notably Japan, Taiwan, and South Korea, have long-established social health insurance systems, poorer countries in the region are still struggling to increase government funding and put basic risk-pooling mechanisms in place to reduce high out-of-pocket payments. For the rest of Asia, social health insurance has been gaining prominence. China and Thailand have made impressive strides towards universal coverage, while Vietnam is working towards the same goal [7–9]. Singapore, known for its medical saving accounts and high private payments, has introduced the Medishield Life, a universal insurance scheme. In contrast to this regional trend, Hong Kong appears the only major economy in East Asia to have a largely unchanged health financing system.

As a matter of fact, Hong Kong has been trying for three decades to diversify its health financing. The repeated failures in doing so clearly reflect the peculiarity of welfare politics in this liberal semi-democracy [10, 11]. Only recently has the Hong Kong Special Administrative Region (SAR) Government decided to launch the Voluntary Health Insurance Scheme (VHIS) in recognition of the fact that no financing arrangement entailing a compulsory contribution would be accepted by the general public. Although private insurance exists in most Asian economies to some extent, Hong Kong’s VHIS stands out as the first scheme in this region to be proposed, promoted, and (in due course) regulated by the government. Many of its features make it a very distinct program, warranting close examination.

*Are Hong Kong citizens willing to subscribe to this voluntary health insurance scheme? Will voluntary insurance succeed in a mixed medical economy?* This study attempts to answer these research questions with empirical evidence. At the time of writing, the public consultation on the VHIS has concluded but the scheme has not yet been launched. Using primary data collected from a telephone survey, this paper analyzes Hong Kong citizens’ responses to the proposed insurance scheme. Such views are of strategic importance due to adverse selection and the other intrinsic problems associated with voluntary insurance. The study identifies a level of demand which is moderate but nonetheless fairly high for a voluntary scheme. Evidence of adverse selection is found, but again of a modest magnitude. Quantitative analysis also identifies and explains the factors associated with respondents’ intention to subscribe. Based on the findings revealed by this study, it is reasonable to be cautiously optimistic about the prospect of the VHIS. Several policy implications for the health financing reforms of Hong Kong and other societies facing similar challenges are also discussed.

### Literature review

Often characterized as voluntary and for-profit commercial coverage, private health insurance actually exists in a variety of forms. Mossialos and Thomson [12] propose a useful classification of private health insurance according to whether it substitutes for the statutory health system, provides complementary coverage for services excluded or not fully covered by the state, or provides supplementary coverage for faster access and increased consumer choice. Referred to as substitutive insurance, the first type exists in Germany, Spain, and the Netherlands where certain groups of people are either not covered by the statutory system or allowed to opt out but free to purchase voluntary health insurance as a substitute [12]. The second type, usually called complementary voluntary health insurance, aims to provide full or partial cover for services that are excluded or not fully covered by the statutory system, such as outpatient and dental care. A substantive complementary health insurance market exists in most OECD countries [4, 5]. In contrast to the substitutive and complementary forms, supplementary health insurance aims to increase consumer choice and improve access to different health services that are usually associated with higher quality and faster access to treatment [4, 12]. Usually tailored to the middle- and high-income strata of the population, supplementary health insurance helps subscribers overcome the inadequacies of the public medical systems, especially long waiting times, and is commonly found in rich societies [5]. As the next section sets out, the proposed VHIS in Hong Kong falls into this latter category.

Voluntary health insurance is prone to various market failures, especially adverse selection and risk selection [13, 14]. Adverse selection occurs when individuals make insurance purchasing decision based on their own assessment of risks or likelihood of making a claim. This phenomenon may result in efficiency losses for both the demand side and supply side, if not properly curbed. Economic theories also identify low risk aversion and moral hazard as important reasons for no or low insurance demand in competitive markets [15]. A large body of literature thus focuses on market demand for voluntary health insurance and the determinants of subscription and willingness to pay. Mossialos, Thomson, and Busse [16] analyze subscriber characteristics in 14 European countries and show that most subscribers to voluntary health insurance come from higher-income groups. Age, gender, occupational status, educational level, and area of residence were also important determinants of willingness to subscribe. Laing and Buisson [17] report that subscribers in the United Kingdom are typically middle-aged professionals, employers, and managers based in London and the South. German subscribers have been found to be predominantly men, younger people, professionals, and those living in western Germany [18]. Coverage in other European countries is similarly dominated by high-income individuals [5, 19]. Related studies were also conducted in the Asian context; Shafie and Hassali, for example, found fairly high level of acceptance among Malaysians to a proposed voluntary community-based health insurance program [20].

Aside from the socioeconomic and demographic factors noted above, dissatisfaction with the public system has also been identified as a key factor behind the purchase of voluntary private health insurance in high-income countries. For instance, long waiting time for National Health Service treatment is often cited as a major factor in the demand for private coverage in the United Kingdom [21, 22]. A strong link between demand and waiting times for elective surgeries in the public system has also been found in Ireland, Australia, and Italy, amongst others [5, 16, 23]; indeed, evidence suggests that voluntary private insurance has enhanced patients’ access to timely hospital care in these systems [5].

Adverse selection constitutes the major challenge to voluntary insurance schemes. There is abundant empirical evidence of this from the US, Australia, China, and Thailand, among many others [24–27]. Various mechanisms to ameliorate adverse selection in the voluntary insurance market have been proposed, including full underwriting, targeted benefit exclusions, waiting periods prior to benefit entitlement, and making the enrolment unit the household instead of individual [28, 29]. However, asymmetric information and practical enforcement difficulties have led to the continued prevalence of adverse selection in many voluntary insurance markets, hampering their further growth. With reference to the findings based on other health systems, this study hypothesizes that the following personal-level characteristics may determine Hong Kong citizens’ willingness to subscribe to the proposed VHIS: gender, age, income, education, health status, status of health insurance subscription, satisfaction with the current health system, and ideological orientation regarding the responsibility of health care.

### The Hong Kong health care system and the reform agenda

**Fig. 1:**
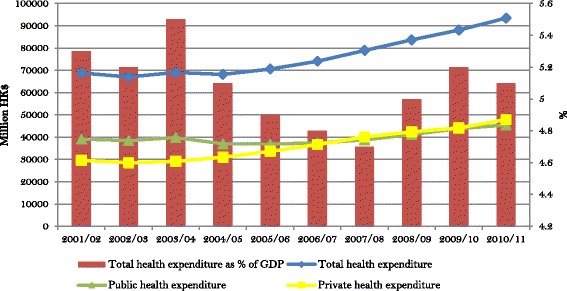
Sources of health financing and total health expenditure as share of GDP, Hong Kong. Note: Total health expenditure is calculated in current market prices. Source: Hong Kong domestic health accounts

**Fig. 2:**
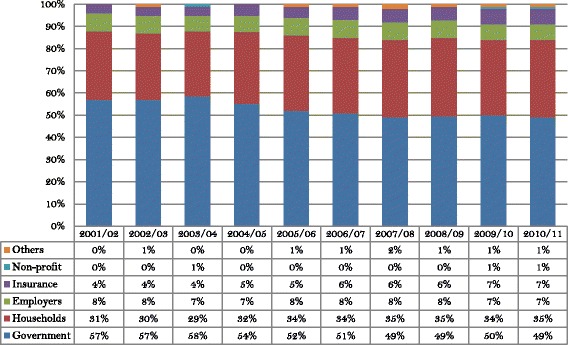
Mix of financing sources of total health expenditure, Hong Kong. Source: Hong Kong domestic health accounts

Hong Kong outperforms most economies on major population health indicators. Life expectancy at birth and the infant and maternal mortality rates are among the best in the world. The health system that created such outstanding performance is financed from both public and private sources, each taking on approximately half the burden (Fig. 1). More than 95% of public funding comes from government tax and nontax revenues with the rest mainly being recovered from fees and charges paid by patients at the point of delivery [30]. Out-of-pocket payments by households form about 36% of total health expenditure. A smaller private contribution to health financing comes from employer-provided group medical benefits and private insurance, accounting for 7.5% and 6.4% of total health expenditure respectively [31]. There is no mandatory health insurance in Hong Kong to date. Fig. 2 shows a breakdown of the sources of health financing. It indicates that household out-of-pocket payment and government subsidies account for the lion’s share while other sources play a rather minor role.

While public facilities dominate secondary and tertiary care in Hong Kong, 70% of primary outpatient services are provided by private clinics. Public hospitals provide close to 90% of inpatient services. Citizens enjoy heavily subsidized services at public facilities without means testing. In the current fee schedule, a general outpatient consultation costs only HK$45 (1 US$ = 7.75 HK$) and a specialist consultation HK$100 for the first attendance, with a HK$10 per item drug charge. Costs for inpatient care are also remarkably low, with a HK$100 per diem charge. Public hospitals are managed by the Hospital Authority (HA), a corporation-like statutory organization answerable to the government. Public hospitals receive over 90% of their income from the HA.

Hong Kong’s health system has achieved excellent performance, with outstanding population health status indicators and high patient satisfaction [32]. However, the system is also confronting daunting challenges. Firstly, rapid demographic change and the epidemiological transition to chronic degenerative diseases have created tremendous pressure on financing. According to government projection, total health expenditure will double by 2030 and further increase to HK$315 billion by 2033. The most alarming message is that public health expenditure as a percentage of total government spending will increase to almost 30% if this trend continues. This may be more than the government can afford [33].

Secondly, low taxes coupled with highly subsidized services give rise to the so-called buffet syndrome. Leung et al. point out that the Hong Kong population is over-reliant on public medical services [30]. In consequence, public facilities are always overloaded. In the outpatient sector, long waiting times as a result of overutilization has been the main problem plaguing the system [34, 35].

The dual challenges of financing and delivery clearly mirror the public-private imbalance of Hong Kong’s health system. On the one hand, the government’s financial responsibility for subsidizing hospital care places a heavy strain on the public purse, whereas private payment is rather minimal. On the other, overwhelmed public facilities are in stark contrast to private facilities which have substantial capacity but are relatively underutilized. The ultimate goal of Hong Kong’s health care reform is thus to recalibrate the public-private mix by reorienting more residents to the private system while leaving the public system to serve the low-income and vulnerable population [33]. Insurance is seen as a good instrument because it can not only mitigate the public-private imbalance in financing, but also divert patient flows through the financial leverage effect [36, 37].

Although social health insurance schemes have been proposed in the past, all reform efforts have failed in the face of tremendous public opposition. It is apparently impossible to impose any compulsory levy from citizens who have become been used to highly subsidized care [10]. In the end, the government had to abandon any reform proposal that would require compulsory individual contributions and proposed a voluntary insurance scheme. The VHIS primarily targets high- and middle-income individuals who can afford and have the desire for greater consumer choice and faster access to services as well as a better quality of services. Its key features include [37]:

- Voluntary subscription of residents;
- Commercial insurance companies as insurers;
- Minimum requirements set by a standard plan that all insurers have to abide by;
- Guaranteed lifetime renewal without re-underwriting;
- No “lifetime benefit limit” as prevalent in the private market;
- Coverage of preexisting conditions subject to a waiting period;
- Benefit coverage to include medical conditions requiring hospital admissions and/or prescribed ambulatory procedures, prescribed advanced diagnostic imaging tests subject to 30% co-insurance, and nonsurgical cancer treatments up to a prescribed limit;
- A high-risk pool to be set up from government revenues to subsidize the premium of subscribers with higher health risks.

## Methods

This study used a telephone survey to investigate Hong Kong citizens’ views of the health financing reforms. Designed by the author, the survey was conducted by the Public Opinion Program of the University of Hong Kong between September 2014 and February 2015. Ethical approval was obtained from the Human Research Ethics Committee of the author’s university. Random-digit dialing was used to select respondents from a computer-generated random-digit dialing pool. As the population of telephone nonsubscribers is rather small in Hong Kong, the coverage error is minimal. The survey targeted Cantonese-speaking adults only, given the very low non-Cantonese speaking population in the city (less than 10%). Weighing adjustment was conducted on age and gender during the process of sampling, based on Census data provided by the Government, so that the sample is able to more accurately reflect key characteristics of the population. Among 2485 successful dials, 1793 respondents accepted the interviews, giving a response rate of 72.15%. Six hundred ninety-two individuals declined our interview; reasons for non-response included unavailability and direct refusal. The profile of respondents is outlined in Table 1. Statistical analysis was performed in STATA 12.0.

**Table 1:** Profile of survey respondents

	Frequency	Percentage
Gender		
Male	695	38.8%
Female	1085	61.2%
Age group		
18–29	222	12.5%
30–39	204	11.5%
40–49	331	18.7%
50–59	432	24.4%
60 and above	583	32.9%
Educational attainment		
Primary and below	259	14.6%
Secondary	800	45.0%
Tertiary and above	719	40.4%
Subjective health status		
Very good	147	8.2%
Good	716	40.0%
Fair	820	45.8%
Bad	82	4.6%
Very bad	26	1.4%
Monthly income		
HK$10,000 and below	852	50.7%
HK$10,001-HK$20,000	524	31.2%
HK$20,001 and above	304	18.1%

Source: author’s survey

The dependent variable of the study is individuals’ intention of subscribing to the VHIS (1 = yes, 2 = no). Explanatory variables include: 1) gender (1 = male, 2 = female), age group (1 = 18–29, 2 = 30–39, 3 = 40–49, 4 = 50–59, 5 = 60 and above), 2) education (1 = primary and below, 2 = secondary, 3 = tertiary), 3) monthly income (1 = <HK$10,000, 2 = HK$10,000–30,000, 3= > HK$30,000), 4) subjective health status (1 = very good, 2 = good, 3 = fair, 4 = bad, 5 = very bad), 5) status of health insurance subscription (1 = uninsured, 2 = insured), 6) satisfaction with Hong Kong’s health system (1 = very dissatisfied, 2 = quite dissatisfied, 3 = half-half, 4 = quite satisfied, 5 = very satisfied). As one’s ideological orientation towards the responsibility of heath care may shape his/her decision in the private insurance market, we measured it with the question “[t]o what extent do you endorse this statement ‘The government should only provide everyone with essential health care services such as care for serious diseases, and encourage people to provide themselves in other aspects’”; answer options included 1 = agree, 2 = half-half and 3 = disagree (variable name = health as private responsibility). For descriptive purpose, the survey instrument also measured respondents’ 1) general view of Hong Kong’s health system, 2) attitudes towards the health financing reform, and 3) reasons for the unwillingness to join the VHIS.

## Results

Respondents were firstly invited to assess Hong Kong’s health system. In the first step, their general views of and satisfaction with the system were measured using a series of qualitative statements. As Table 2 shows, while approximately half of the respondents held positive views, demand for major reform was also fairly strong.

**Table 2:** Respondents’ general views of and satisfaction with Hong Kong’s health care system

	Frequency	Percentage
Opinion on the way in which the health care system of Hong Kong runs		
* On the whole, the health care system of Hong Kong is run well.*	260	15.3%
* There are some good things in the way the health care system of Hong Kong is run, and only minor changes would make it work better.*	575	33.9%
* There are some good things in the way the health care system of Hong Kong is run, but only fundamental changes would make it work better.*	670	39.5%
* The health care system of Hong Kong is run so badly that we need to rebuild it completely.*	191	11.3%
Total	1696	100%
Satisfaction with the health care system of Hong Kong		
Very satisfied	68	3.9%
Quite satisfied	733	42.0%
Half-half	585	33.5%
Quite dissatisfied	270	15.5%
Very dissatisfied	91	5.2%
Total	1747	100%

Source: author survey

Secondly, and more importantly, respondents were invited to indicate their attitudes towards the reform. More than half (52.3%, *N* = 816) supported it, and about one third (36.7%, *N* = 512) expressed an intention to subscribe. The reasons for non-subscription were explored. Important reasons cited included “already insured in the private market and no intention to switch” (26.5%), “high premium” (26.1%), and “no need” (14.8%).

Multivariate analyses were then used to examine the factors associated with having an intention to subscribe, which is a binary variable. Logistic regressions results are presented in Table 3, with the odds ratios and confidence intervals reported. Based on the conclusions of studies conducted in other health systems, age, education, income, gender, and health status were first included as potential explanatory variables. Model 1 suggests that higher income and educational attainment are significantly associated with intention to subscribe, which is consistent with the findings of previous studies.

**Table 3:** Results of logistic regression analysis

	Model 1		Model 2		Model 3	
Monthly income						
<HK$10,000	1		1		1	
HK$10,000–30,000	1.600***	[1.186,2.159]	1.599***	[1.174,2.179]	1.617***	[1.182,2.214]
>HK$30,000	1.732***	[1.180,2.541]	1.639**	[1.102,2.436]	1.629**	[1.090,2.433]
Educational attainment						
Primary and below	1		1		1	
Secondary	1.703***	[1.154,2.512]	1.547**	[1.042,2.296]	1.544**	[1.021,2.333]
Tertiary	1.874***	[1.217,2.887]	1.686**	[1.088,2.614]	1.801**	[1.143,2.838]
Age						
18–29	1		1		1	
30–39	0.835	[0.511,1.365]	0.76	[0.461,1.254]	0.755	[0.456,1.252]
40–49	1.095	[0.701,1.712]	0.979	[0.620,1.545]	0.931	[0.587,1.475]
50–59	1.670**	[1.092,2.551]	1.551**	[1.006,2.390]	1.507*	[0.973,2.332]
60 and Above	1.304	[0.859,1.979]	1.243	[0.811,1.905]	1.212	[0.787,1.865]
Sex						
Male	1		1		1	
Female	0.838	[0.658,1.067]	0.854	[0.668,1.093]	0.868	[0.675,1.118]
Self-reported health status						
Very good	1		1		1	
Good	1.12	[0.716,1.754]	1.197	[0.752,1.904]	1.274	[0.794,2.042]
Fair	1.255	[0.801,1.966]	1.408	[0.883,2.246]	1.454	[0.904,2.337]
Bad	0.914	[0.448,1.864]	1.035	[0.501,2.140]	1.051	[0.499,2.215]
Very bad	2.131	[0.825,5.504]	2.659*	[0.993,7.120]	3.461**	[1.229,9.752]
Satisfaction with health system						
Very dissatisfied			1		1	
Quite dissatisfied			1.557	[0.836,2.900]	1.556	[0.809,2.992]
Half-half			1.536	[0.854,2.761]	1.560	[0.839,2.899]
Quite satisfied			1.801**	[1.010,3.210]	1.689*	[0.915,3.118]
Very satisfied			2.249**	[1.008,5.018]	2.118*	[0.923,4.861]
Insured?						
No			1		1	
Yes			1.369**	[1.055,1.778]	1.361**	[1.043,1.776]
Health as private responsibility						
Disagree					1	
Half-half					1.429*	[0.966,2.115]
Agree					1.559***	[1.203,2.021]
*Pseudo R2*	0.0302		0.0377		0.0461	
*N*	1309		1278		1237	

Exponentiated coefficients; 95% confidence intervals in parentheses; * *p* < 0.10, ** *p* < 0.05, *** *p* < 0.01

It was also found that those aged between 50 and 59 demonstrated significant willingness to subscribe, reflecting some degree of adverse selection. Interestingly, there was no significant association between intention to subscribe and membership of the oldest age group (aged 60 and above: consistent across all models). This can be explained by the fact that being older leads to a higher premium which may undermine senior citizens’ interest. More importantly, the universal coverage of Hong Kong’s public medical system coupled with minimum fees makes health care highly accessible even to the very poor. It would not be surprising to find that those in poor health conditions and older people are already in the public medical system. A piece of supporting evidence comes from Chan et al. [38] who note that overall, patients attending private hospitals in Hong Kong had less chronic illnesses and were generally in better health compared with those who had opted for public facilities. This is arguably due to lower costs and high levels of patient trust. As a result of the high satisfaction with the public system and the higher costs that would be incurred in private facilities, it is hardly surprising that older adults display little motivation to switch. In contrast, those belonging to the age group 50–59 are typically still in the labor force, have comparatively higher ability to pay, and are increasingly concerned with health. Gender was not significant in any of the models. Overall, Model 1 explains 3.02% of the variance in the dependent variable.

In Model 2, respondents’ satisfaction with the public system and insurance status were included in the regression analysis. While all explanatory variables identified in Model 1 remained statistically significant, people with very bad health status demonstrated stronger interest in subscription; the level of significance further increased in Model 3 after ideological orientation had been controlled. As clear evidence of adverse selection, this also suggests citizens in poor health have a desire for faster access to services, especially specialty care and major procedures, given the long waiting time in the public system. Model 2 improves the model fit by 25% and explains approximately 3.77% of the variance in the dependent variable, while Model 3 further increases the percentage to 4.61%.

Both Models 2 and 3 suggest that those who are already insured in the private market are more likely to subscribe or switch to the VHIS. This might initially appear puzzling, because it is usually expected that people without any insurance coverage tend to have a stronger desire for risk aversion and insurance protection. However, in a health system where every citizen is guaranteed equal access to government-provided health care, a lack of insurance may not create much motivation for subscription. Putting it simply, Hong Kong’s public hospital system has been working “too well” to motivate people to move away from it. The insured population, who are economically better off and more inclined towards risk aversion, may have the desire to subscribe or switch to a supplementary policy and to enjoy greater flexibility in choosing providers.

Most studies in the Western context find dissatisfaction with the health system to be an important reason for the purchase of supplementary private insurance. However, this study shows that higher satisfaction is actually a significant predictor of intention to subscribe in Hong Kong. This can be explained by the fact that satisfaction reflects not only personal experiences of seeking care, but also overall confidence with the system as a whole. In other words, potential subscribers do not need to worry about quality of care or other issues when switching providers. With the public system serving essentially as a “safety net” for everyone, satisfied patients may have more interest in using the private system for flexibility and choice.

Ideological orientation towards health care was controlled for in Model 3. While the results from Models 1 and 2 remained robust, unsurprisingly it was found that those who viewed health as primarily a private responsibility demonstrated significantly higher willingness to join the voluntary insurance scheme. Overall, the random nature of sampling and weighting adjustment based on gender and age have ensured that the sample demographically matched to the Hong Kong population, thus strengthening the external validity of this study.

## Discussion

The role of voluntary private health insurance is increasingly recognized in both developing and developed societies. Struggling to correct the public-private imbalance in its health care system, the Hong Kong SAR Government seeks to introduce a government-regulated voluntary health insurance scheme. Its main purpose is to relieve the heavy pressure on the public medical system by reorienting the economically better off to the private sector. Such insurance may be appealing to this segment of the population because of their desire for increased consumer choice, faster access to care, and more personalized services.

Reflecting the government’s attempt to tackle these daunting health policy challenges, the VHIS is a very new and distinctive financing instrument that combines the characteristics of private insurance with strong government regulation. This study analyzed citizens’ responses to the new scheme. A telephone survey of 1793 randomly sampled Hong Kong adults demonstrates a fairly high level of satisfaction with the health system, although many respondents believed that reform is necessary. Slightly more than one third of the respondents explicitly stated their intention of subscribing to the VHIS. This figure is fairly high considering the scheme’s voluntary nature.

The analysis also revealed moderate evidence of adverse selection. People in higher-risk categories may not necessarily flock to the pool as many of them have already been using the city’s highly subsidized public medical services and may not want to move away from these. A key component of the VHIS is a high-risk pool subsidized by the government, which can partly absorb the effect of adverse selection. However, it appears that the government’s generosity is of limited interest to the high-risk population. Thirdly, people without insurance did not demonstrate as significant a level of interest as might have been anticipated, implying that the highly equitable and accessible public medical services in Hong Kong essentially act as a safety net for citizens and therefore a lack of insurance is not a significant concern, especially for those on low incomes. Finally, satisfaction with the public medical system was found to be associated with stronger willingness to purchase the voluntary insurance. This suggests that in a health system with high levels of public satisfaction, particularly in terms of equity and cost, people’s confidence in the entire system becomes a major source of the motivation to support government-led policy reforms.

## Conclusions

This study identifies three policy implications for health financing reforms in Hong Kong and other health systems encountering similar challenges. Firstly, voluntary insurance could play a helpful role in broadening the base of health care financing and correcting the public-private imbalance, provided that there are both substantive market demands and a competent private system through which to render services. Government regulation is essential if key social policy goals are to be achieved. Secondly, market demands for private insurance are closely related to citizens’ satisfaction with the public system. The excellent performance of Hong Kong’s public health care system in terms of equity, accessibility, and cost has had two parallel impacts. On the one hand, high-risk residents, particularly the uninsured, do not face a pressing need to switch out of the overloaded public system despite its inadequacies; this, in turn, may reduce the impact of adverse selection. On the other hand, high satisfaction reinforces the interests of those who have both the need for services and the ability to pay for supplementary insurance. Thirdly, and for these reasons, the high-risk population (that is, the heavy users of the public system) demonstrates a moderate interest in the insurance despite the availability of government subsidies. This may offset the effect of the reform to some extent. Evidence from Australia suggests that premium rebate or tax benefit had merely yielded limited outcome in encouraging private insurance participation. How similar incentives would work in Hong Kong merits more in-depth investigations [39].

Overall, this study indicates cautious optimism for the financing reform as far as the participation rate is concerned; after all, one third of respondents expressed an interest in subscribing, a considerably high percentage for a voluntary scheme. However, its long-term health and sustainable development relies on the effective containment of costs, the maintenance of affordable premium, and the proper regulation of private providers.

## Abbreviations

Hong Kong SAR: Hong Kong Special Administrative Region
OECD: Organization for Economic Co-operation and Development
VHIS: The Voluntary Health Insurance Scheme
